# Optimizing Reactive Responses to Outbreaks of Immunizing Infections: Balancing Case Management and Vaccination

**DOI:** 10.1371/journal.pone.0041428

**Published:** 2012-08-10

**Authors:** Petra Klepac, Ottar N. Bjørnstad, C. Jessica E. Metcalf, Bryan T. Grenfell

**Affiliations:** 1 Department of Ecology and Evolutionary Biology, Princeton University, Princeton, New Jersey, United States of America; 2 Center for Infectious Disease Dynamics, The Pennsylvania State University, University Park, Pennsylvania, United States of America; 3 Fogarty International Center, National Institute of Health, Bethesda, Maryland, United States of America; 4 Department of Zoology, University of Oxford, Oxford, United Kingdom; 5 Woodrow Wilson School, Princeton University, Princeton, New Jersey, United States of America; Yale University, United States of America

## Abstract

For vaccine-preventable infections, immunization generally needs to be supplemented by palliative care of individuals missed by the vaccination. Costs and availability of vaccine doses and palliative care vary by disease and by region. In many situations, resources for delivery of palliative care are independent of resources required for vaccination; however we also need to consider the conservative scenario where there is some trade-off between efforts, which is of potential relevance for resource-poor settings. We formulate an SEIR model that includes those two control strategies – vaccination and palliative care. We consider their relative merit and optimal allocation in the context of a highly efficacious vaccine, and under the assumption that palliative care may reduce transmission. We investigate the utility of a range of mixed or pure strategies that can be implemented after an epidemic has started, and look for rule-of-thumb principles of how best to reduce the burden of disease during an acute outbreak over a spectrum of vaccine-preventable infections. Intuitively, we expect the best strategy to initially focus on vaccination, and enhanced palliative care after the infection has peaked, but a number of plausible realistic constraints for control result in important qualifications on the intervention strategy. The time in the epidemic when one should switch strategy depends sensitively on the relative cost of vaccine to palliative care, the available budget, and 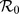. Crucially, outbreak response vaccination may be more effective in managing low-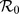 diseases, while high 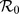 scenarios enhance the importance of routine vaccination and case management.

## Introduction

In addition to direct protection of individuals, a key aim of routine vaccination programs is to reach population levels of immunity sufficient to prevent epidemics by breaking the chain of transmission (‘herd immunity’) [1]. If one can immunize prophylactically beyond herd immunity, palliative care is not necessary for improving the population's health; where this is not the case, and if resources for palliative care and vaccination are independent, both should be administered as required. Timing may be a component of this: in low immunization coverage rate areas (e.g. resource-poor setting such as sub-Saharan Africa), vaccination campaigns sometimes have to operate ‘reactively’ after major epidemics have begun (outbreak response vaccination) [2]–[6], and need to be supplemented by palliative care of cases missed by the campaign. In developing countries, resources for controlling infectious diseases are often limited, providing additional constraints on the logistics of outbreak control. In this paper we develop theory to understand the case where resource limitations impose a trade-off between the timing and the extent of vaccination and palliative care.

While vaccination strategies have been extensively studied, e.g. [1], [3], [6]–[11], the combination of vaccination with palliative care has not to our knowledge been considered systematically (although the treatment alone has been considered by [12]–[15]; also see [16], [17]). Here we focus on both vaccination and case management as reactive responses to manage outbreaks of immunizing infections. We use simple strategic models to explore the optimal patterns of control delivery across a range of epidemiological contexts. Specifically, we ask: *Are there other strategies we can implement after the epidemic has started? What is the optimal balance between preventing infection by reactive vaccination and directly treating disease in infected individuals? Can we find the best possible strategy given the various logistical and economic constraints that may reflect low-income settings, and how does it depend on the timing of control delivery?*

We start by considering disease progression during an unvaccinated epidemic and explore general effects of vaccination and palliative care using a simple continuous time SEIR model (Susceptible Exposed Infectious Removed). To model relative allocation of immunizations and palliative care for different diseases, we used a discrete time model approach as this facilitates partitioning of resources. Our analysis is driven by the pragmatics of World Health Organization (WHO) guidelines for managing acute outbreaks [3], [5], [18], and we investigate the impact of current outbreak response measures – including case management and immunization campaigns, and assessing outbreak alert thresholds. This initial work leaves out various complexities, such as age structure, spatial heterogeneities, and pathogen immune escape, which we return to in the discussion.

## Models

### Unconstrained model

**Figure 1:**
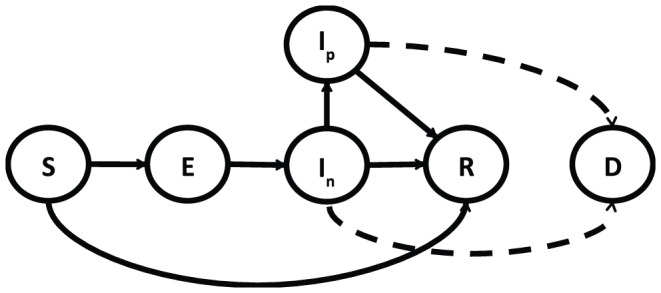
SEIR model with two types of control, vaccination and palliative care. Infected individuals that receive palliative care form a separate compartment 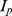; vaccination moves individuals from the S compartment to the R compartment, both untreated (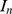) and treated (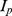) infected individuals may move into mortality compartment (D), according to their respective case fatality rates.

To study the relative merits of the different interventions for controlling acute immunizing infections, we use the simplest model that captures the essence of the balance between immunization and palliative care. We start with the epidemiological Susceptible, Exposed, Infectious and Removed (SEIR) model [19]–[22] and modify it to include the two options of epidemic control: vaccination and palliative care (Figure 1). Susceptible individuals are vaccinated at a rate 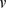 (for simplicity we assume here 100% vaccine efficacy). Palliative care - treating infectious cases - may reduce the rate at which individuals transmit the disease and increase the rate at which they recover, so the model has two infectious classes to distinguish between infectious cases that get palliative care (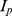) from those that do not (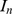). The case fatality rates for the two classes are 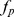 and 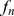 respectively. Assuming frequency dependent transmission [23]–[28] the model is embodied in the following system of equations:
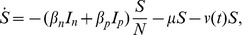
(1a)

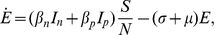
(1b)

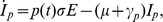
(1c)

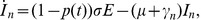
(1d)

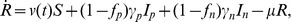
(1e)

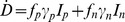
(1f)where 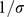 is the duration of the latent period, births are balancing deaths at the rate 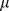, 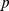 is the proportion of cases that get palliative care treatment, and the average duration of infection with or without palliative care is given by 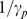 and 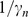, respectively.

Note that we consider a rapid epidemic, so we ignore all host demography except disease induced mortality. Individuals who are sick enough to seek treatment are likely to be hospitalized or kept at home, and will thereby be isolated to a certain degree from the general population. This can lead to reduced rate of transmission of the treated class (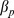) compared to the non-treated one (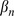) so we allow that 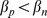. We also assume that treatment of infectious cases leads to reduced fatality rate, so 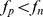.

Rather than focusing on the prevention of infection and reduction in the number of cases, as in [29]–[31] we define the success of a strategy in terms of the lives saved compared to an unmanaged outbreak. We consider the effectiveness of reactive response measures during the time course of an outbreak, and look for the optimal combination and timing of case treatment vs vaccination. The best strategy minimizes the number of fatal cases, that is, it maximizes the proportion of lives saved compared to an unmanaged outbreak.

We base model (1) on measles dynamics [6], [32]–[38] but later explore a larger parameter space over a range of 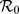 values, and discuss implications of our results for other immunizing infections.

### Constrained model

To implement logistic constraints into the model, we discretize from rates to transition probabilities assuming piecewise constant rates, and a time step equal to one day.
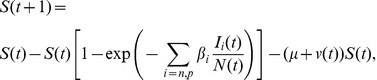
(2a)

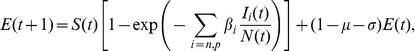
(2b)

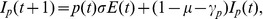
(2c)

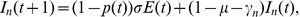
(2d)

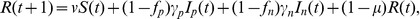
(2e)

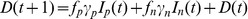
(2f)


To investigate the balance of timing and the optimal allocation of limited resources in managing an outbreak we assume there is a fixed budget 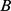 available to control the epidemic, a proportion of which is allocated to palliative care (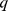) and the rest to immunizations (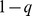). When 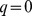 all of the resources are invested in immunizations, and when 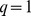 all of the budget is allocated to the treatment of cases. For mixed strategies (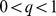), both control methods are used simultaneously. Immunizations and palliative care are administered until the budget runs out. Once implemented, a control strategy is difficult to change at short notice, so we identify the best static control strategy, as in, e.g. [39]–[41]. In addition to the available budget, the total number of administered doses also depends on the per capita costs of vaccine dose (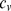) and for one unit of palliative care (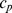). We assume that it is harder to find infected or susceptible individuals when there are fewer of them, so we let the number of individuals that receive a treatment be proportional to their abundance, up to certain thresholds for the number of individuals that can be vaccinated (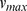) and treated (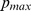) in a day. More specifically, vaccination and palliative care are administered according to:
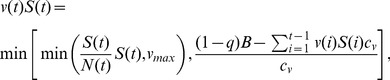
(3)

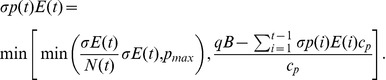
(4)


We find the optimal allocation of resources by choosing a value of 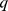 that maximizes the proportion of lives saved compared to the unmanaged outbreak, as defined by the objective function 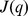, and subject to the epidemic trajectories given in (1).
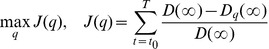
(5)The time 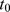 is the time of control delivery during an outbreak and is determined by the epidemic threshold alert (the point in the outbreak when a certain number of cases is confirmed and reported). The control is administered either until the budget runs out, or until the end of the outbreak at time 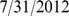.

If the logistic constraints are such that we can either only vaccinate, or only administer palliative care at a given time (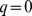 or 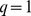, respectively) we look for the optimal time in the outbreak to switch between the two strategies (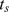).
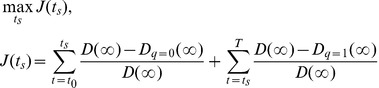
(6)The unique optimal values of these deterministic models are found numerically in Matlab.

To capture variation in transmission and the rate of increase of the epidemic, we perform the same analysis over a range of 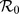 values. Since the duration of the outbreak is shorter for larger 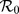 we rescale the time with respect to the epidemic and align the inflection points and the peaks of the epidemic trajectories, to be able to compare different response strategies (see Figure S1 for illustration). We use 3 different values of budget 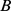 in simulations: (i) limiting budget (not enough to vaccinate everyone), (ii) enough budget to vaccinate everyone, if entire budget is spent on vaccination (but not enough for both vaccination and palliative care), (iii) and a large budget (enough for both vaccination and palliative care). Ratios of costs per unit vaccine (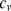) and unit palliative care (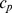) vary between: (i) 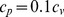, (ii) 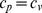, (iii) 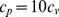, (iv) 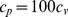.

## Results

**Figure 2:**
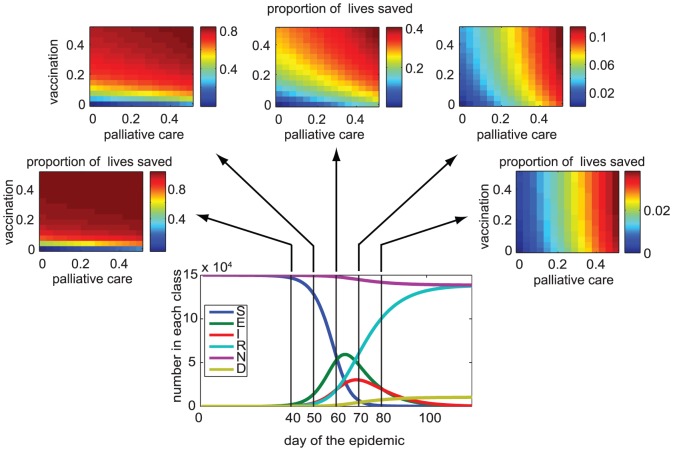
Proportion of lives saved compared to the baseline case over the range of intensities of vaccination (y-axis) and palliative care (x-axis) in simulated epidemics for different times of onset of control implementation. Red colors represent higher effectiveness. Bottom graph shows the epidemic curves for the uncontrolled case; the vertical lines show the days of the outbreak for which we implement control (vaccination and palliative care) in relation to the peak of the epidemic. The model is parameterized according to the 2003–2004 measles outbreak among children in Niamey. Parameter values: 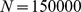, 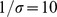 days, 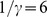 days, 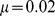 year^−1^, 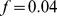, 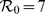.

To establish how effective different combinations of vaccination and palliative care are at reducing mortality, we parameterize the model using data from the 2003–2004 measles outbreak in Niger, and consider a range of control scenarios implemented at different times in the outbreak: 40 days, 50 days, 60 days, 70 days and 80 days after the beginning of the outbreak (see Figure 2 and Figure S1 for an illustration of timing of control relative to the peak of the outbreak). The trajectories of an unmanaged outbreak are shown on the lower panel; the colored surfaces show the proportion of lives predicted to be saved over the range of intensities of vaccination and palliative care in simulated epidemics. At the beginning of an outbreak, immunization is the more effective strategy of curtailing the outbreak (the color gradient on the corresponding surface is predominantly horizontal) whereas case-management has more impact on the population level after the epidemic has peaked (vertical color gradient on the corresponding surfaces) because vaccine-enhancement of protection through herd immunity is greatly diminished after this inflection point. Note, however, that in the latter case the proportion of lives saved is much smaller (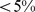 reduction in mortality) than for the early intervention strategies (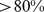 reduction in mortality). Importantly, reactive vaccination can make a significant impact even if the campaign is started 60 days after the outbreak showing that outbreak response vaccination campaigns can significantly reduce mortality in ongoing epidemics where prophylactic vaccination is not possible.

**Figure 3:**
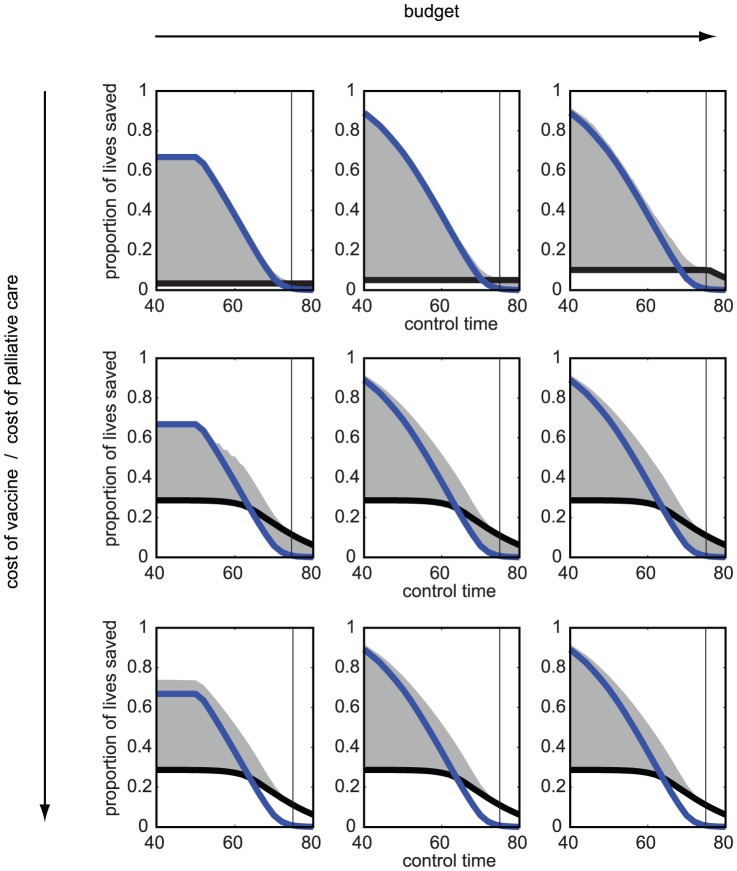
Proportion of lives saved compared to the case where control is implemented. Blue line shows the vaccination-only strategy, black line shows case-management-only strategy, and gray shows a range of mixed strategies where both vaccination and palliative care are used simultaneously and the budget is split the two, vertical line shows where the infection peaks. Columns from left to right show increasing budget values, whereas rows show increasing per capita cost of vaccine to palliative care ratio (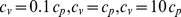). Columns show increasing budget levels (from left to right: 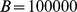, 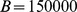, 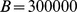 dimensionless units of cost). 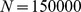, 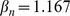, 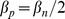, 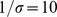 days, 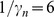 days, 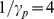 days, 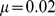 year^−1^, 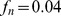, 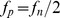, 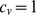.

While Figure 2 illustrates the unconstrained case (either strategy can be adopted, with no limits on investment), Figure 3 compares the outcomes of possible strategies in a constrained model corresponding to 3 different budget levels and a range of relative costs of vaccination and palliative care. When palliative care is expensive compared to vaccination (Figure 3 first row) vaccination is the best strategy, even late in the outbreak. In the presence of logistic and resource constraints, palliative care becomes effective on the population level as it becomes affordable relative to the vaccine cost (Figure 3, bottom row). On the population level, the outcome of implementing a control strategy on mortality does not qualitatively change with the available budget, although, as expected, more cases can be treated with larger budget.

**Figure 4:**
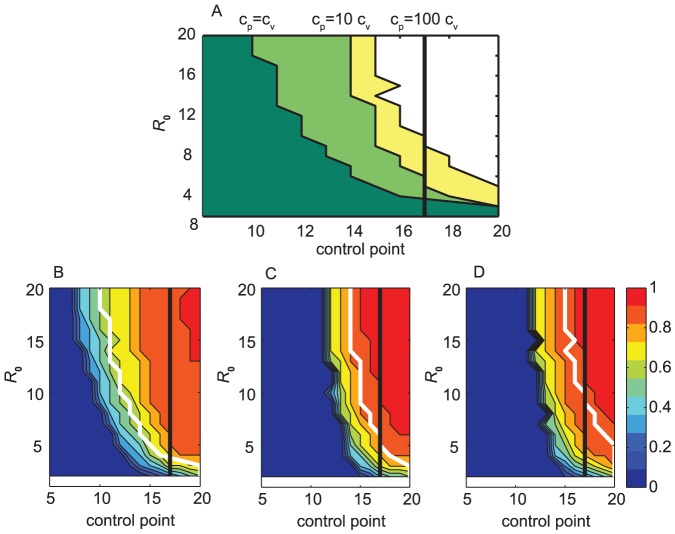
Optimal strategies over a range of 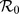
 **values.** (A) The time in the outbreak when the strategy should be switched from vaccine-only to a palliative-care-only strategy, for 3 ratios of costs of unit palliative care and unit vaccine: 1, 10, 100 (curves from left to right, dark green, light green, and yellow, respectively). Time is rescaled to epidemic time (in relation to the duration of the epidemic that changes with 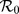) so that outbreaks for all values of 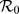 peak along the vertical black line. (B)–(D) Best performing strategy over a range of 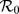 values and different times of control. The colorbar shows the proportion of the budget invested in palliative care; 0 (dark blue) is vaccination-only strategy, 1 (dark red) is palliative-care-only strategy. Red colors correspond to palliative-care-intense strategies, and strategies in the blue region focus on vaccination. Time is rescaled to the epidemic so that for all 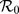 values the epidemic peaks along the black line. The white line shows the time at which one should switch from a vaccine-only to a palliative-care-only strategy in (A). In (B) the cost of per unit palliative care (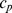) is equal to per unit cost of vaccine (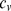), 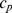 = 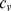; (C) 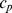 = 10 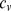; (D) 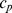 = 100 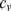. Limited budget (100000 cost units). Outbreak alert threshold is set to 10 cases. Parameters: 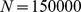, 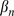 and 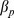 are varied according to 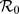 such that 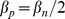, 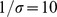 days, 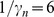 days, 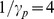 days, 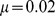 year^−1^, 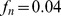, 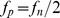, 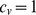.

The effectiveness of outbreak response vaccination even late in the outbreak is also evident in the presence of constraints modeled in (2). If the logistic constraints are such that only one type of control (either vaccination or palliative care) can be delivered at a time, Figure 3 shows it is best to focus on vaccination-only strategy (blue line) early in the outbreak, and palliative-care-only strategy late in the outbreak (black line). The timing in the outbreak where it is best to switch between these two strategies is at the intersection of pure strategies (where black and blue lines cross). Figure 4 shows the optimal switching time 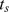 for a range of 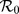 values for three different ratios of unit vaccination and palliative care costs (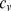 and 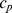, respectively). The optimal time to switch would be expected to be when the epidemics peak, but for high-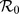 values switching should occur before the peak, especially when palliative care is affordable. This is because the infection spreads faster than we can contain it with immunizations when 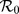 is high, and case management becomes important in reducing overall case-fatality rate, though vaccination early in the outbreak always out-performs palliative care (the dark green area on Figure 4A).

If the infection spreads very quickly (large values of 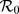), the window of opportunity to curtail the outbreak with vaccination becomes very narrow (blue area in Figure 4B–D) and vaccination is quickly supplemented with palliative care (Figure 4B–D, red regions). When resources are limited, and both vaccine and palliative care can be administered simultaneously, vaccine-intense strategies are most effective early in the outbreak, and remain effective throughout for low 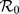 values where vaccination can occur faster than the disease spreads. For high-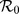 diseases timing is key - vaccination is effective only very early in the outbreak; preference is given to mixed strategies and case management even before the epidemics peak. Uncertainty in the epidemic parameters doesn't qualitatively change the results, but pushes the administration of palliative-care to a slightly earlier time point (though parameter and structural uncertainty can change the optimal control more substantially in other systems (Figure S3), as in [42]).

## Discussion

In an ideal world, there should never be a trade-off between the extent and timing of palliative care and vaccination. There would be enough vaccine doses for everyone (ideally given prophylactically before epidemics), with infrastructure that ensured rapid delivery; furthermore all infected cases who were not immunized would get appropriate and timely treatment. However, vaccination sometimes needs to be given reactively, and, in some circumstances there may be a trade off between vaccination and palliative care. Here we try to identify general rules for how to best make those decisions in the worst case scenarios of constraints between different interventions.

We use a SEIR-type model for acute immunizing infections inspired by measles epidemiology, and explore it over a wide range of epidemiological parameters thereby encompassing other infections such as mumps, rubella, influenza. The model is a simple one, without any heterogeneity in transmission, demography or space, but our preliminary analysis provides important insight into the efficacy and robustness of common reactive control strategies: palliative care and vaccination. In the simplest analysis without budget constraints, outbreak response vaccination is an effective way of reducing mortality and morbidity, especially if vaccination starts early in the outbreak. As a general rule one should vaccinate as much as possible early in the outbreak, and then switch to palliative care after the infection has peaked.

In the presence of budget limitation and logistic constraints (e.g. uncertainty in outbreak detection, or delays in control introduction) the best strategy is inconsistent with that expected in the simplest case. For high 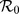 palliative care becomes optimal even before the peak; and for low 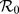 palliative care may not be particularly beneficial when implemented after the peak. The more affordable the palliative care, the more effective it becomes earlier in an outbreak up to a limit of vaccine efficacy - early in an outbreak, vaccine is always most effective, since the number of cases we can avert by vaccination is larger than the number of deaths prevented with case management. For high 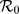 diseases the rate of spread of the infection soon becomes faster than the rate of immunization - reactive vaccination is no longer an effective way of controlling an outbreak in this case, and the focus should be on routine vaccination and palliative care.

Uncertainty in the timing of the outbreak, or introduction of the control measures later in the outbreak slightly shifts the optimal strategy to a mixed palliative care and vaccination strategy, and then to an entirely palliative care strategy after the epidemic has peaked. At the beginning of the outbreak it is unlikely that a true value of 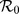 is known. Here we have presented the simplest case. In practice, a number of intricacies will complicate the picture; for example, age and contact-structure, e.g. [7], [43]–[45], space [33], [46], [47], recurrent epidemics and pathogen life history. Since our focus is on managing a single outbreak, our epidemic model does not include a discount factor for the value of future infections. Discount rates can change the nature of the optimal strategy for disease control [12], [48], [49] and as such should be considered for determining long-term control strategies.

For small 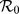 infections and perfect vaccine, vaccination alone is enough to interrupt the chain of transmission and contain the outbreak. Seasonal influenza has a low effective reproduction ratio of infection, 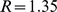 [50], so we would expect immunizations programs alone to be the best strategy for control. However, in reality, the flu vaccine is imperfect and the virus adapts to escape prevailing immunity, so that multiple vaccinations are required for protection. This can lead to variation in repeat vaccine efficacy because of differences in antigenetic distances among vaccine strains and between the vaccine strains and the epidemic outbreak strain [51]. Such reductions in vaccine efficacy will put more importance on indirect protection via palliative care.

In cases where the detection methods fail to recognize the outbreak early, the opportunity to interrupt the transmission with the direct and indirect protection offered by vaccination is missed, so the best control strategy is intense case management (palliative care) even at low 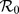. This emphasizes that a key issue in successful outbreak control is early detection and rapid response [52]. Improving detection methods and lowering the threshold for outbreak alert to only a few infected cases is essential for transmission interruption. For logistic reasons there are certain delays associated with outbreak responses. Lowering threshold alerts to just one detected case for measles (as suggested by WHO Communicable disease profile for Niger, [53]) could provide indispensable time that would allow control measures to be implemented early enough to stop transmission.

Operational realities are inevitably more complex than the framework presented here, and in particular, the source of budgets for vaccination vs. palliative care may be rather different, and thus not reflect the trade-off implemented here. However, overall, our results provide a strategic view of how information on timeliness and budget and logistic constraints, as well as characteristics of the infection, like 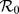, affect the most effective intervention for immunizing infections.

## Supporting Information

Figure S1
**Cumulative number of cases for 3 different values of** 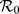 **: 1.5, 3, and 15.** Shaded areas represent the areas of epidemic trajectory between the threshold alert (10 infected cases) and the inflection point after the peak of infection (dashed lines) Đ the considered time interval for control interventions. In Figure 4 in the manuscript we rescale the time so that for all 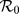 values the first control point refers to the time the epidemic trajectory has crossed the alert threshold, the last control point is the inflection point after the peak, and for all 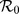 values the epidemic peaks at the same point. The narrowness of the window of opportunity for 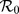 = 15 provides a far lower opportunity for reaching herd immunity, in addition to the fact that herd immunity in this case requires far more individuals to be vaccinated. Parameters: 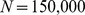, 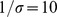 days, 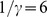 days, 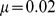 year^−1^, 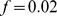.(TIF)

Figure S2 **Rescaled version of** **Figure 2** **in the manuscript using the same colorbar scale for all the subplots.**
(TIF)

Figure S3
**Best performing strategy over a range of** 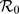 **values and different times of control shown in color.** Red colors correspond to palliative-care-intense strategies, and strategies in the blue region focus on vaccination. The colorbar shows the proportion of the budget invested in palliative care; 0 (dark blue) is vaccination-only strategy, 1 (dark red) is palliative-care-only strategy. For all 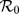 values the epidemic peaks along the black line. The white line shows the time at which one should switch from vaccine-only to palliative-care-only strategy. Epidemic threshold alert is set to 10 cases; limiting budget; 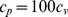. (A) 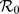 value is fixed (assumed to be known). (B) Uncertainty in 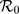 represented by a range of values (uniform distribution, range 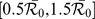, where 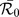 is the mean value).(TIF)
