# Supplementary figures and images for: Optimizing Reactive Responses to Outbreaks of Immunizing Infections: Balancing Case Management and Vaccination
Source: PLoS One. 2012 Aug 10;7(8):e41428. doi: 10.1371/journal.pone.0041428 (PMC3416818; doi:10.1371/journal.pone.0041428)

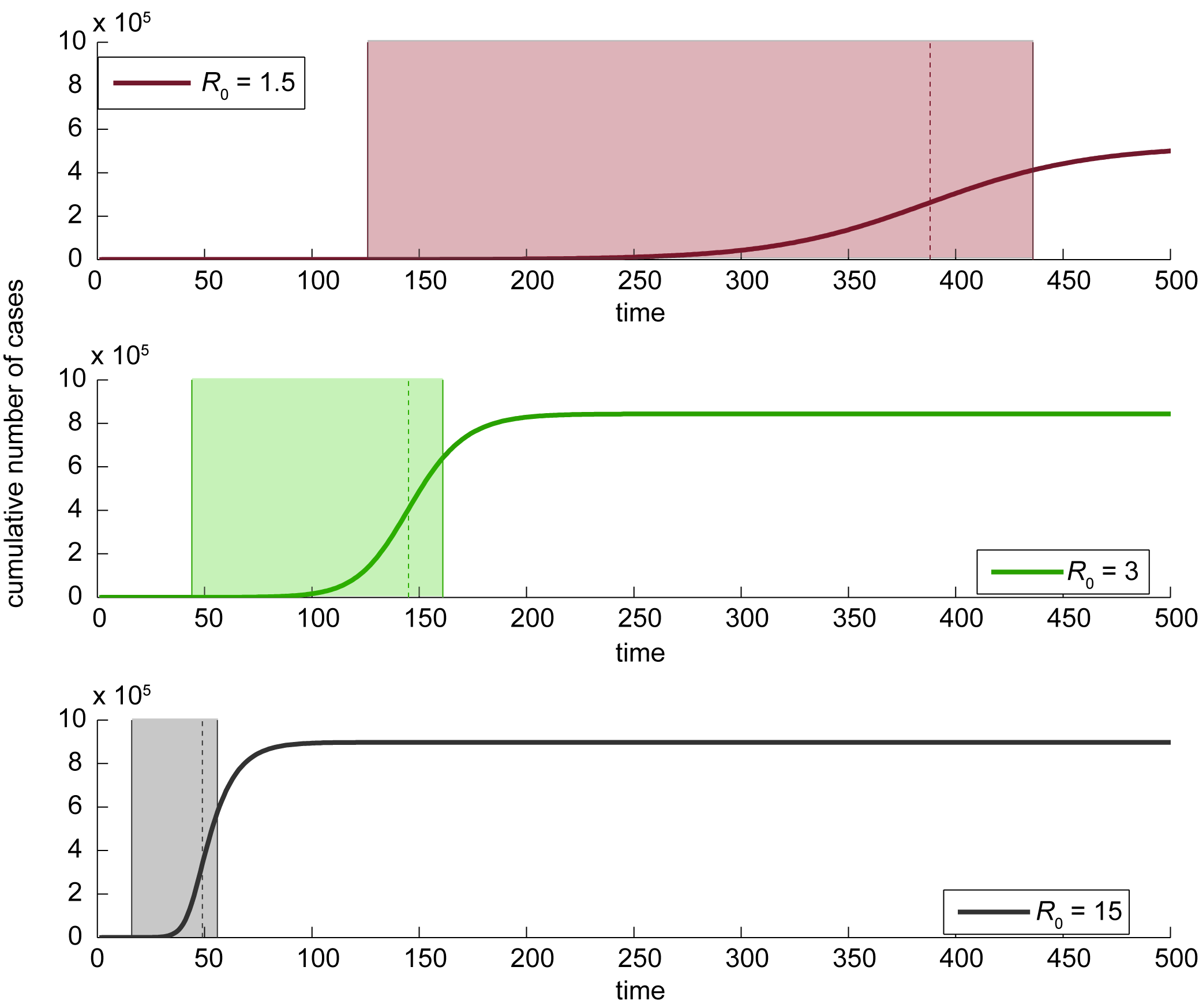

Supplement: Figure S1 — Cumulative number of cases for 3 different values of : 1.5, 3, and 15. Shaded areas represent the areas of epidemic trajectory between the threshold alert (10 infected cases) and the inflection point after the peak of infection (dashed lines) Đ the considered time interval for control interventions. In Figure 4 in the manuscript we rescale the time so that for all values the first control point refers to the time the epidemic trajectory has crossed the alert threshold, the last control point is the inflection point after the peak, and for all values the epidemic peaks at the same point. The narrowness of the window of opportunity for = 15 provides a far lower opportunity for reaching herd immunity, in addition to the fact that herd immunity in this case requires far more individuals to be vaccinated. Parameters: , days, days, year−1, . (TIF) [file pone.0041428.s001.tif]

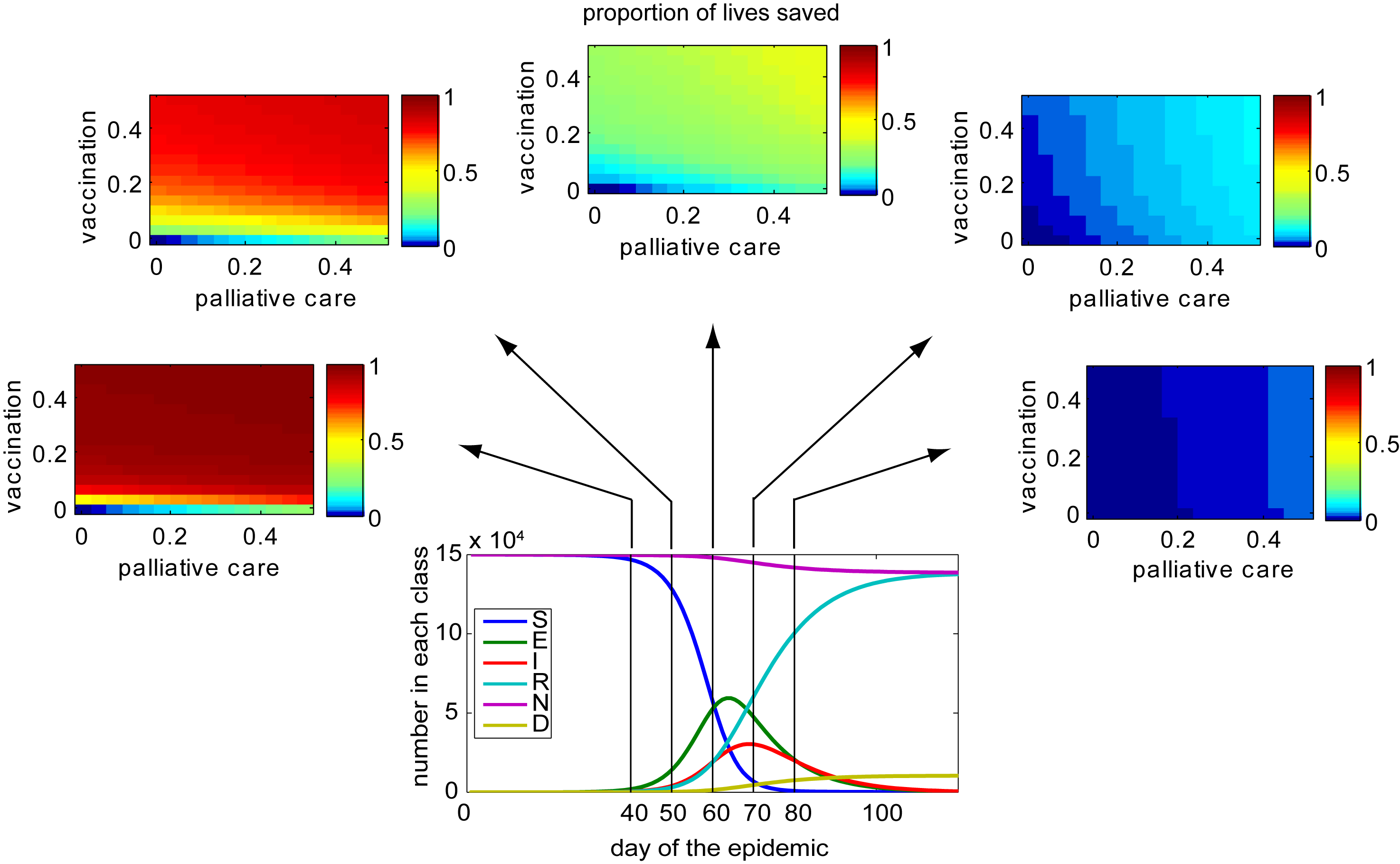

Supplement: Figure S2 — Rescaled version of Figure 2 in the manuscript using the same colorbar scale for all the subplots. (TIF) [file pone.0041428.s002.tif]

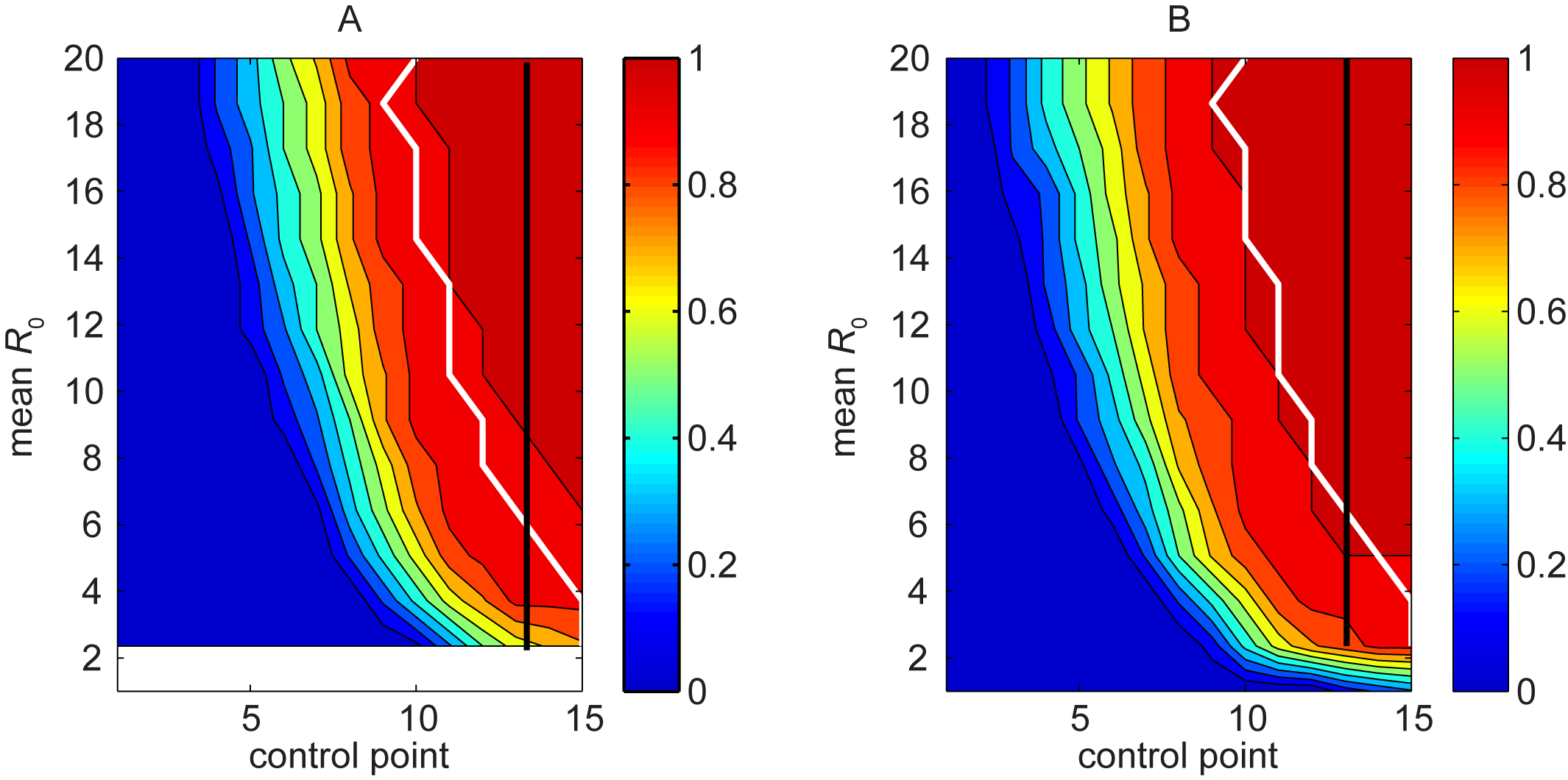

Supplement: Figure S3 — Best performing strategy over a range of values and different times of control shown in color. Red colors correspond to palliative-care-intense strategies, and strategies in the blue region focus on vaccination. The colorbar shows the proportion of the budget invested in palliative care; 0 (dark blue) is vaccination-only strategy, 1 (dark red) is palliative-care-only strategy. For all values the epidemic peaks along the black line. The white line shows the time at which one should switch from vaccine-only to palliative-care-only strategy. Epidemic threshold alert is set to 10 cases; limiting budget; . (A) value is fixed (assumed to be known). (B) Uncertainty in represented by a range of values (uniform distribution, range , where is the mean value). (TIF) [file pone.0041428.s003.tif]
